# Inhibition Within the Lateral Habenula—Implications for Affective Disorders

**DOI:** 10.3389/fnbeh.2021.786011

**Published:** 2021-11-26

**Authors:** Jack F. Webster, Salvatore Lecca, Christian Wozny

**Affiliations:** ^1^Strathclyde Institute for Pharmacy and Biomedical Sciences, Strathclyde University, Glasgow, United Kingdom; ^2^The Department of Fundamental Neurosciences, The University of Lausanne, Lausanne, Switzerland; ^3^MSH Medical School Hamburg, IMM Institute for Molecular Medicine, Medical University, Hamburg, Germany

**Keywords:** lateral habenula, major depressive disorder, inhibition, local inhibitory interneurons, inhibitory afferents

## Abstract

The lateral habenula (LHb) is a key brain region implicated in the pathology of major depressive disorder (MDD). Specifically, excitatory LHb neurons are known to be hyperactive in MDD, thus resulting in a greater excitatory output mainly to downstream inhibitory neurons in the rostromedial tegmental nucleus. This likely results in suppression of downstream dopaminergic ventral tegmental area neurons, therefore, resulting in an overall reduction in reward signalling. In line with this, increasing evidence implicates aberrant inhibitory signalling onto LHb neurons as a co-causative factor in MDD, likely as a result of disinhibition of excitatory neurons. Consistently, growing evidence now suggests that normalising inhibitory signalling within the LHb may be a potential therapeutic strategy for MDD. Despite these recent advances, however, the exact pharmacological and neural circuit mechanisms which control inhibitory signalling within the LHb are still incompletely understood. Thus, in this review article, we aim to provide an up-to-date summary of the current state of knowledge of the mechanisms by which inhibitory signalling is processed within the LHb, with a view of exploring how this may be targeted as a future therapy for MDD.

## Introduction

The lateral habenula (LHb) is a brain structure within the epithalamus which is now well established to have a causative role in the pathogenesis of major depressive disorder (MDD; Sartorius et al., 2010; Li et al., 2011; Yang et al., 2018a; Hu et al., 2020). Long-range excitatory neurons in the LHb project to and exert net inhibitory control over the ventral tegmental area (VTA; Ji and Shepard, 2007; Jhou et al., 2009), and the dorsal raphe nucleus (DRN; Wang and Aghajanian, 1977; Ferraro et al., 1996), thus acting as the “off switch” of the midbrain reward circuitry. From an evolutionary standpoint, this serves the important function of ensuring behaviours with negative outcomes are not reinforced (Hikosaka, 2010). However, this system becomes dysregulated in MDD and the LHb becomes hyperactive (Li et al., 2011; Lecca et al., 2016; Tchenio et al., 2017; Cui et al., 2018; Yang et al., 2018a), thus likely potentiating inhibitory modulation of the downstream reward circuitry and curtailing the associated positive emotions.

In addition to the wealth of information that now exists relating to how the LHb controls its’ efferent targets, it is also known that the LHb receives afferent input from a variety of brain regions pivotal in emotional processing. These include the internal segment of the globus pallidus (Hong and Hikosaka, 2008), analogous to the rodent entopeduncular nucleus (Shabel et al., 2014; Meye et al., 2016; Wallace et al., 2017), the lateral hypothalamus (Stamatakis et al., 2016; Lecca et al., 2017; Lazaridis et al., 2019; Trusel et al., 2019) and the ventral pallidum (Knowland et al., 2017; Faget et al., 2018; Stephenson-Jones et al., 2020; Pribiag et al., 2021), as well as receiving reciprocal input from the VTA (Stamatakis et al., 2013; Root et al., 2014). These structures provide a combination of excitatory, inhibitory and GABA/glutamate co-releasing input to the LHb, in a very fine balance which has been shown to be altered in depressive states (Shabel et al., 2014; Knowland et al., 2017). In general, convergent evidence suggests that excitatory LHb afferents promote aversive states and depressive behaviour (Barker et al., 2017; Knowland et al., 2017; Lecca et al., 2017; Lazaridis et al., 2019), while conversely inhibitory afferents promote behavioural reinforcement (Faget et al., 2018; Stephenson-Jones et al., 2020) and reduce depressive behaviour (Winter et al., 2011; Huang et al., 2019). Taking this into account, we here review the current state of knowledge regarding the processing of inhibitory signalling within the LHb. We will discuss what is currently known about inhibitory input to the LHb, how this is altered in depressive states, and how this may eventually be exploited to develop novel therapies for MDD.

## Inhibitory Afferents of The LHb

### The Globus Pallidus Internal Segment/Entopeduncular Nucleus

The globus pallidus internal segment, or the analogous entopeduncular nucleus (EP) in rodents, is one of the primary afferent structures to the LHb. This structure is thought to be primarily GABAergic in nature (Oertel et al., 1984; Stephenson et al., 2005), although the EP-LHb pathway has a net excitatory effect on postsynaptic LHb neurons (Shabel et al., 2012; Stephenson-Jones et al., 2016). As such LHb-projecting EP neurons are thought to primarily encode aversion (Stephenson-Jones et al., 2016; Li H. et al., 2019). However, a striking feature of these neurons is that they co-release GABA and glutamate at LHb synapses (Shabel et al., 2012, 2014; Wallace et al., 2017; Lazaridis et al., 2019). This co-release phenomenon has been shown to be shifted in favour of reduced GABA in a rodent model of depression, and conversely towards increased GABA following antidepressant treatment (Shabel et al., 2014). Similarly, EP-LHb projections are known to be involved in cocaine withdrawal (Meye et al., 2016) and avoidance (Li H. et al., 2021), with a shift towards reduced GABA observed during cocaine withdrawal (Meye et al., 2016), thus consistent with the hypothesis that enhanced excitatory input to the LHb promotes aversive states. Consistently, potentiation of glutamatergic signalling at the EP to LHb synapse has also been observed in another rodent model of depression, with both an increase in presynaptic glutamate release probability and an increase in postsynaptic AMPA receptor expression thought to be causative mechanisms (Cerniauskas et al., 2019). Hence, the current evidence surrounding the functionality of LHb-projecting EP neurons appears to point to a scenario whereby the balance of glutamate and GABA release is in a fine balance which is shifted in favour of glutamate in aversive states, thus potentiating excitatory drive onto LHb neurons.

### The Basal Forebrain

A basal forebrain is a group of heterogenous forebrain structures, composed of GABAergic, glutamatergic and cholinergic neurons (Zaborszky et al., 2012). Within the basal forebrain, the primarily GABAergic (Root et al., 2015) ventral pallidum (VP) is known to be another major LHb afferent. LHb-projecting VP neurons however are known to be distinct populations of both excitatory and inhibitory neurons (Faget et al., 2018; Stephenson-Jones et al., 2020), although recent work has also identified VP neurons that express both markers of GABAergic and glutamatergic neurons, and may co-release GABA and glutamate at LHb synapses (Pribiag et al., 2021). As with the EP, glutamatergic LHb-projecting VP neurons are activated by negative stimuli (Stephenson-Jones et al., 2020) and encode aversion (Faget et al., 2018; Tooley et al., 2018; Stephenson-Jones et al., 2020). Consistently, these neurons become more excitable in a rodent model of depression (Knowland et al., 2017), and ablating these glutamatergic neurons promotes reward-seeking behaviour (Tooley et al., 2018). Conversely, GABAergic LHb-projecting VP neurons are excited by reward-predicting stimuli and promote reward-seeking (Stephenson-Jones et al., 2020). These results seem to indicate that the VP-to-LHb projection exists as somewhat of a push-pull system, whereby inhibitory and excitatory LHb-projecting VP neurons oppositely promote reward and aversion, respectively.

However, while the VP-to-LHb projection is the most well-studied, other basal forebrain regions are believed to project to and modulate LHb activity. One study found that LHb-projecting GABAergic neurons within the basal forebrain as a whole promote aggressive behaviour, which is believed to be rewarding and is thus consistent with the hypothesis that GABAergic LHb afferents promote reward (Golden et al., 2016). Furthermore, another study identified a population of neurons in the portion of the basal forebrain ventral to the VP which projects to the LHb and expresses the GABAergic marker VGAT (Zhu et al., 2017), although the functionality of these neurons specifically at LHb synapses was not elucidated. Another work has identified a GABAergic projection to the LHb from the diagonal band of Broca which has a role in regulating hippocampal theta rhythm (Aizawa et al., 2013). Thus, it is becoming increasingly clear that the basal forebrain appears to be a major source of inhibitory input to the LHb, with various populations of LHb-projecting neurons encoding a variety of behavioural functionalities.

### Other Inhibitory Afferents to the LHb

The LHb also receives GABAergic innervation from the VTA (Stamatakis et al., 2013; Root et al., 2014). Similarly to LHb-projecting neurons within the EP, VTA neurons have intriguingly been shown to co-release GABA and glutamate at LHb synapses (Root et al., 2014). This pathway appears to be capable of bidirectionally controlling LHb activity (Root et al., 2014), possibly indicative of a feedback loop whereby hypo or hyperactivity within the LHb may be limited by reciprocal VTA connectivity. In addition to these co-releasing neurons, a further population of LHb-projecting VTA neurons has also been identified which unusually expresses the classical dopaminergic marker tyrosine hydroxylase, but exclusively releases GABA within the LHb (Stamatakis et al., 2013). While it remains unclear whether or not these neurons are indeed dopaminergic (Lammel et al., 2015), they are consistent with other GABAergic LHb afferents in that they encode reward (Stamatakis et al., 2013).

The lateral preoptic area (LPO) of the hypothalamus also contains distinct populations of excitatory and inhibitory neurons which provide convergent input to individual LHb neurons (Barker et al., 2017). These populations of neurons appear to control the LHb in a manner similar to the VP in that excitatory LPO neurons encode aversion, while inhibitory LPO neurons promote reward. Yet intriguingly, both of these populations respond to aversive stimuli (Barker et al., 2017). Additionally, GABAergic afferents from the lateral hypothalamus have also been identified (Stamatakis et al., 2016; Lecca et al., 2017), although these are fairly minimal in comparison to excitatory drive from this region.

Emerging evidence has also pointed to inhibitory LHb afferents arising from the thalamus. A projection to the LHb arising from the ventral lateral geniculate nucleus and intergeniculate leaflet of the thalamus has recently been shown to be critically involved in the antidepressant effects of light therapy (Huang et al., 2019). We have also recently described a population of neurons within the mediodorsal thalamus nucleus which inhibits the LHb (Webster et al., 2020), although the behavioural influence of this pathway remains to be clarified.

**Figure 1:**
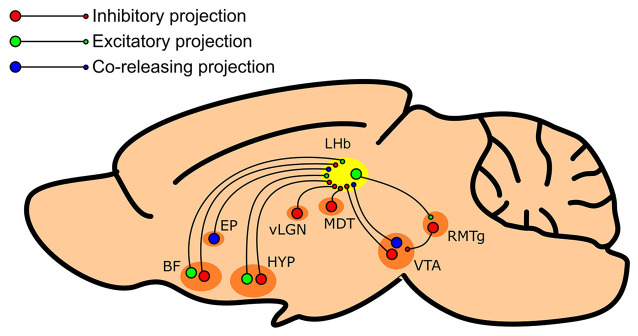
Summary schematic of regions which provide inhibitory input to the LHb. The LHb (shown in yellow) receives inhibitory input from various regions such as the entopeduncular nucleus (EP), the basal forebrain (BF), the lateral preoptic area and lateral hypothalamus of the hypothalamus (HYP), the ventral lateral geniculate nucleus/intergeniculate leaflet complex of the thalamus (vLGN), the mediodorsal thalamus (MDT), and the ventral tegmental area (VTA). These afferent structures may feature both inhibitory (shown in red) and excitatory (shown in green) neuronal populations, primarily inhibitory populations, or GABA/glutamate co-releasing neurons (shown in blue).

In summary, there is now much evidence indicating that the LHb receives afferent inhibitory innervation from a wide variety of sources (Figure 1). However, regardless of the source, there appears to be remarkable consistency within the literature; that is that inhibitory innervation of the LHb encodes reward, while excitatory innervation encodes aversion.

## Locally-Targeting Inhibitory LHb Neurons

It is well-accepted that the majority of LHb neurons form a physiologically homogenous, but morphologically diverse population (Weiss and Veh, 2011), of which most neurons are glutamatergic (Omelchenko et al., 2009; Brinschwitz et al., 2010). Yet mounting evidence now indicates the existence of locally-targeting inhibitory neurons (Zhang et al., 2018; Flanigan et al., 2020; Webster et al., 2020; Nakamura et al., 2021), which may form multiple distinct sub-classes. One such population express the inhibitory marker glutamic acid decarboxylase 2 (GAD-2) and are confined to the medial LHb (Flanigan et al., 2020). These neurons have been shown to inhibit other LHb neurons to promote aggressive behaviour in mice (Flanigan et al., 2020) which, as mentioned earlier, can be interpreted as a reward-seeking behaviour (Golden et al., 2016). We and others have recently shown that some LHb neurons which express the well-known cortical interneuron marker parvalbumin (Tremblay et al., 2016) are GABAergic and inhibit local LHb neurons (Webster et al., 2020; Nakamura et al., 2021). These neurons are likely distinct from the aforementioned population of GAD-2 expressing neurons in that they are confined to the lateral LHb and also express the vesicular GABA transporter (VGAT; Webster et al., 2020), another well-known marker of inhibitory neurons, but largely do not express GAD-2 (Nakamura et al., 2021). Note however that the expression patterns of GABAergic markers within the LHb appears to differ between mice and rats (Quina et al., 2020), with VGAT expression in rats observed in the medial LHb and GAD-2 expression largely absent (Zhang et al., 2018; Quina et al., 2020). Furthermore, early electrophysiological and morphological studies have proposed that a population of neurons akin to the very distinctive cortical neurogliaform interneuron exists within the LHb in rats (Weiss and Veh, 2011; Wagner et al., 2016), although these neurons appear to be different to their cortical counterparts at least in that they do not express similar molecular markers (Webster et al., 2021).

**Figure 2:**
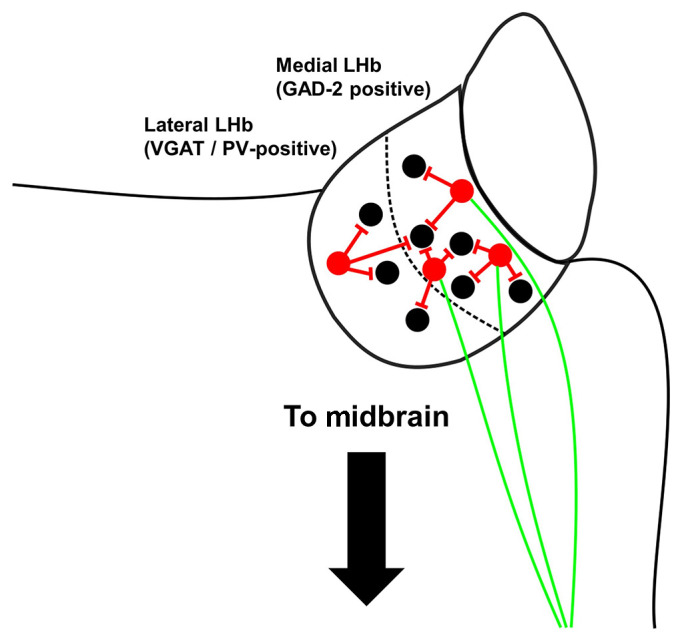
Summary schematic of local inhibitory neurons within the mouse LHb. Locally-targeting inhibitory neurons (shown in red) in the medial LHb express glutamic acid decarboxylase 2 (GAD-2) but not vesicular GABA transporter (VGAT) and may also have excitatory projections to the tegmentum (shown in green), although note that Flanigan et al. (2020) found no evidence that inhibitory GAD-2-positive neurons project outside the LHb. Inhibitory neurons in the lateral LHb express VGAT and parvalbumin, but most do not express GAD-2.

Interestingly, however, some studies have also shown that LHb neurons which express inhibitory markers also express the excitatory marker vesicular glutamate transporter 2 (VGLUT-2), and project to the midbrain (Zhang et al., 2018; Quina et al., 2020). Furthermore, these neurons appear to release exclusively glutamate in the tegmentum of the midbrain (Quina et al., 2020). Thus it may be the case that within the LHb, inhibitory neurons do not exist exclusively as the well-described populations of locally-targeting interneurons that they are known to form in other brain structures (Tremblay et al., 2016), but rather may be dual-functioning neurons which serve the additional purpose of exciting downstream regions (Figure 2).

## Modulation of LHb Inhibitory Signalling by Other Neurotransmitters and Neuropeptides

While the majority of work studying the role of the LHb in MDD and other psychiatric disorders has focused on alterations in direct excitatory and inhibitory inputs, there is an emerging body of evidence indicating that various other neurotransmitters and neuropeptides can influence the activity of the LHb, both directly and indirectly. These are discussed below.

### Orexin

Interestingly, orexin receptor 2 expression within the LHb appears to be largely confined to GABAergic neurons (Zhang et al., 2018; Flanigan et al., 2020), hence suggesting that orexinergic modulation of the LHb may act *via* GABAergic neurons. At least some of these neurons are the same aforementioned GAD-2 expressing LHb neurons, which have been shown to be activated by orexinergic input from the lateral hypothalamus to promote aggressive behaviour (Flanigan et al., 2020). These neurons also express receptors for vasopressin, serotonin, and dopamine (Zhang et al., 2018), and as such may respond to a variety of transmitters. It is interesting to note that chronic social defeat stress (CSDS) also appears to activate orexinergic inputs to the LHb in socially defeated mice (Wang et al., 2021). Furthermore, direct infusion of orexin into the LHb alleviates the depressive phenotype induced by CSDS (Wang et al., 2021). Thus, current evidence seems to indicate that orexinergic signalling within the LHb is generally associated with social interactions between dominant and subordinate mice, but may be multifunctional; specifically, this can promote aggressive phenotypes in dominant mice, and bidirectionally control depressive behaviour in subordinate mice.

### Endocannabinoids

Other recent work has pointed to the role of endocannabinoid signalling in modulating LHb function (Shepard and Nugent, 2021). CB1 receptor activation has been shown to reduce inhibitory synaptic input onto LHb neurons (Authement et al., 2018), while at the behavioural level, intra-LHb infusion of Δ9-tetrahydrocannabinol has been shown to induce a deficit in impulsivity control in rats (Zapata and Lupica, 2021).

### Opioid Receptors

Opioid receptor signalling has also recently been shown to modulate neuronal excitability within the LHb (Simmons et al., 2020). Interestingly, Kappa opioid receptor activation bidirectionally excites and inhibits LHb neurons based on the size of the hyperpolarization-activated cation currents the neurons express. This is accompanied by a net decrease in both excitatory and inhibitory synaptic release onto LHb neurons (Simmons et al., 2020). Thus it may be the case that dynorphin/Kappa opioid receptor signalling has opposing effects on differing sub-populations of LHb neurons. However, the origin of preprodynorphin/dynorphin-positive fibres targeting LHb neurons remains unknown (Chen et al., 2020).

### Cholinergic Signalling

While the role of cholinergic signalling within the adjacent medial habenula in nicotine withdrawal and drug addiction has been relatively well-studied in recent years (Lee et al., 2019), a specific role for cholinergic signalling within the LHb has also recently started to become apparent (Zapata et al., 2017; Wolfe et al., 2021). In a manner similar to Kappa opioid receptor activation, cholinergic receptor activation appears to differentially excite or inhibit different populations of LHb neurons, while also suppressing both excitatory and inhibitory synaptic inputs; although this effect appears to more strongly suppress excitatory input hence resulting in an overall net shift towards increased inhibition (Wolfe et al., 2021).

### Neuropeptide Y

Neuropeptide Y (NPY) has recently been shown to modulate both excitatory and inhibitory synaptic transmission within the LHb (Cheon et al., 2019). Specifically, NPY receptor activation appears capable of bidirectionally modulating excitatory input in different groups of neurons, and has a net reduction of inhibitory input mediated by Y1 receptors (Cheon et al., 2019, 2020).

### Serotonin and Dopamine

It has long been known that the LHb acts as a modulator of midbrain dopaminergic and serotonergic systems. However, some evidence indicates that these transmitters can also modulate the activity of the LHb. Serotonin has been shown to reduce both excitatory and inhibitory transmission at the entopeduncular nucleus to LHb synapses (Shabel et al., 2014). Conversely, dopamine appears to excite a subset of LHb neurons in a D4 receptor-dependent manner, possibly indicative of an excitatory feedback loop connecting the LHb with the ventral tegmental area (Good et al., 2013).

Thus, an emerging image appears to be forming whereby other signalling systems are capable of modulating LHb activity. Although the behavioural relevance of these inputs remains largely unclear, it is interesting to note that many of these transmitters appear capable of both exciting and inhibiting different populations of LHb neurons. Therefore, it may be the case that these transmitters are targeting distinct neuronal sub-populations, and as such future work which differentiates these sub-populations based on the expression of receptors for such transmitters may serve to unveil much novel information regarding how the LHb encodes behaviour.

## Inhibition of The LHb as A Potential Therapy for Depression

To date, the majority of work focusing on the LHb as a potential therapeutic target for MDD has aimed to reduce the excitability of LHb neurons (for reviews, see Nuno-Perez et al., 2018; Yang et al., 2018b; Hu et al., 2020). Referring back to the original hypothesis that the LHb is hyperactive in depression (Li et al., 2011), there is now substantial evidence that reducing the excitability of LHb neurons *via* a variety of means has an antidepressant effect. Much work has focussed on normalising LHb activity by the use of deep brain stimulation (DBS), a technique whereby electrodes are implanted within the brain to modulate neuronal activity (Sartorius et al., 2010; Meng et al., 2011; Tchenio et al., 2017; Jakobs et al., 2019). This technique appears to hold great promise as a therapeutic intervention in that LHb-targeted DBS has been shown to induce full remission in a human patient (Sartorius et al., 2010). Interestingly, the antidepressant effect of DBS appears to be dependent on the stimulation frequency, in that high frequency stimulation (>100 Hz) has an antidepressant effect in rodents (Meng et al., 2011; Tchenio et al., 2017; Jakobs et al., 2019), while lower frequencies stimulation (5–20 Hz) appears to promote depressive behaviour (Elmer et al., 2019; Jakobs et al., 2019).

But what are the mechanisms by which high frequency DBS within the LHb elicits an antidepressant effect? Interestingly, in a recent investigation in rodents, Tchenio et al. (2017) showed that firing activity of LHb neurons recorded *in vivo* decreases during local LHb DBS. The authors, in line with a previous study (Li et al., 2011) also demonstrated that such high frequency stimulation dampens AMPA transmission onto LHb cells. It has also been proposed that DBS activates presynaptic GABAergic terminals to promote GABA release (Li et al., 2004), which may be a possible explanation given the extensive GABAergic innervation the LHb receives (Brinschwitz et al., 2010). However, whether DBS modulates GABAergic transmission in the LHb remains to be tested.

Modulating the electrical firing pattern and synaptic activity of LHb neurons *via* various pharmacological interventions has also been shown to have therapeutic benefits. For instance, blockade of NMDA receptors reduce bursting activity, and is at least partially responsible for the antidepressant actions of ketamine (Cui et al., 2018; Yang et al., 2018a), while AMPA receptor blockade has also been demonstrated to have antidepressant efficacy (Li et al., 2017; Zhang et al., 2019). Additionally, both local LHb chemogenetic inhibition (Nair et al., 2013; Tchenio et al., 2017), and direct pharmacological inhibition (Winter et al., 2011) and input-specific synaptic inhibition (Huang et al., 2019) have displayed efficacy as potential therapeutic options in rodent models of depression. The therapeutic benefits of direct inhibition of the LHb can be linked to both GABA_A_and GABA_B_ signalling. Direct injection of the GABA_A_ agonist muscimol has been shown to alleviate depressive symptoms in both the classical learned helplessness rat model of depression (Winter et al., 2011) and interestingly in depression induced by a rat model of Parkinson’s disease (Wang et al., 2017). Moreover, foot shock exposure in mice triggers a diminished functionality in GABA_B_ transmission and has been observed in various rodent models of depression, such as acute learned helplessness (Lecca et al., 2016), maternal separation (Tchenio et al., 2017), and chronic social defeat (Li Z.-L. et al., 2021). Altogether the latter studies highlight the GABA_B_ receptor in the LHb as a potential target to treat depressive symptoms. Mechanistically, a stressful event (i.e., foot shock exposure) in mice triggers internalisation of both GABA_B_ receptors and G protein-gated inwardly rectifying potassium (GIRK) channels, along with a concurrent increase in protein phosphatase 2A activity, which is known to regulate the expression of these channels. Consistently, pharmacological inhibition of protein phosphatase 2A normalises GABA_B_and GIRK expression and alleviates depressive symptoms (Lecca et al., 2016). It is also notable that glycinergic signalling within the LHb has been linked to depressive behaviour, in that direct injection of glycine has an anxiolytic effect in a rat model of alcohol withdrawal, while intra-LHb injection of the glycinergic antagonist strychnine is anxiogenic (Li W. et al., 2019).

## Concluding Remarks

In summary, as the mechanisms by which inhibitory signalling within the LHb are processed become clearer, so too do the potential strategies by which this can be exploited as a therapeutic strategy in MDD. The consistency between pre- and postsynaptic modulation of LHb neurons in relation to depressive behaviour is striking- inhibitory afferents promote reward-like states, while excitatory afferents promote aversion, and consistently activation of postsynaptic inhibitory GABA and glycine receptors appears to have an antidepressant effect, as does blockade of excitatory AMPA and NMDA receptors. As such, novel therapies for MDD may attempt to capitalise on this knowledge by aiming to selectively modulate such pathways.

## Author Contributions

All authors contributed to the article and approved the submitted version.

## Conflict of Interest

The authors declare that the research was conducted in the absence of any commercial or financial relationships that could be construed as a potential conflict of interest.

## Publisher’s Note

All claims expressed in this article are solely those of the authors and do not necessarily represent those of their affiliated organizations, or those of the publisher, the editors and the reviewers. Any product that may be evaluated in this article, or claim that may be made by its manufacturer, is not guaranteed or endorsed by the publisher.
